# ARDS following oesophagectomy: a comparison of two trials

**DOI:** 10.1136/bmjresp-2017-000207

**Published:** 2017-11-02

**Authors:** Phillip A Howells, Kerrie A Aldridge, Dhruv Parekh, Daniel Park, Olga Tucker, Rachel C A Dancer, Fang Gao, Gavin D Perkins, David R Thickett

**Affiliations:** 1 Peri-operative and critical care trials group, Institute of Inflammation and Ageing, University of Birmingham, Birmingham, UK; 2 Department of Respiratory Medicine, University Hospitals Birmingham NHS Trust, Birmingham, UK; 3 Department of Critical Care Medicine, Heart of England NHS Foundation Trust, Birmingham, UK; 4 Department of Upper Gastrointestinal Surgery, Heart of England NHS Foundation Trust, Birmingham, UK; 5 Warwick Clinical Trials Unit, Warwick Medical School, University of Warwick, Birmingham, UK

**Keywords:** ards, tobacco and the lung, drug induced lung disease

## Abstract

**Introduction:**

The Beta Agonist Lung Injury Trial-Prevention (BALTI-P) translational substudy and Vitamin D to Prevent Acute Lung Injury Following Oesophagectomy (VINDALOO) trials recruited patients undergoing oesophagectomy, 4 years apart. The acute respiratory distress syndrome (ARDS) rates were lower in the VINDALOO trial. We sought to identify changes between these two trials and identify risk factors for ARDS in oesophagectomy.

**Methods:**

There were data available from 61 patients in the BALTI-P substudy and 68 from VINDALOO. Databases were available for both trials; additional data were collected. Multivariate logistic regression was used to analyse risk factors for ARDS and postoperative complications in the cohorts combined.

**Results:**

Logistic regression analysis showed active smoking was associated with an increase in ARDS (OR 3.91; 95% CI 1.33 to 11.5) and dihydropyridine use (OR 5.34;95% CI 1.56 to 18.3). Hospital length of stay was longer for those who took dihydropyridines (median 29 days (IQR 17–42) vs 13 days (IQR 10–18), P=0.0007) or were diabetic (median 25 days (IQR 14–39) vs 13 (IQR 10–19), P=0.023) but not for current smokers (median in never/ex-smokers 13 (IQR 10–23) vs current smokers 15 (IQR 11–20), P=0.73).

**Conclusions:**

Smoking cessation trials should be promoted. Dihydropyridine effects perioperatively require further clinical and mechanistic evaluation. Patients undergoing oesophagectomy are a useful model for studying perioperative ARDS.

**Key messages**

- ARDS following oesophagectomy is associated with adverse outcome.
- Smoking and dihydropyridines are associated with postoperative acute respiratory distress syndrome (ARDS) in oesophagectomy. Although the cohort appears to be changing, oesophagectomy remains a useful clinical model of ARDS.

## Introduction

Patients undergoing oesophagectomy have high rates of postoperative complications^1^ including the acute respiratory distress syndrome (ARDS).^2^ We have previously shown that ARDS following oesophagectomy is associated with more non-respiratory organ failure, longer critical care and hospital stays,^3^ and other groups have demonstrated worse short-term and long-term outcomes associated with ARDS^2^ and other pulmonary complications.^4^ Severe infection and cardiac dysrhythmias are common.^5–7^ However, this high complication rate, alongside the planned nature of surgery and the clear timing of the surgical insult, makes oesophagectomy a potentially useful model to undertake trials to reduce perioperative complications.^8^

Both the Beta Agonists in Lung Injury Trial-Prevention (BALTI-P),^9^ which completed recruitment in 2011, and the Vitamin D to Prevent Acute Lung Injury Following Oesophagectomy (VINDALOO) trials, completed in 2015,^10^ used oesophagectomy as a model of ARDS. We observed that the incidence of ARDS in the VINDALOO (8 out of 68, 11.8%) cohort was substantially lower than in the BALTI-P (83 out of 331, 25.1% and 14 out of 61, 23%) substudy (see the Methods section below), independent of a pharmacological effect of the agents trialled, suggesting that there had been changes between the groups that were expected a priori to be similar.

The aims of this work were to determine which clinical features were different between the two cohorts that might explain the differences in postoperative ARDS and complications. The combined cohorts were analysed to seek further risk factors not apparent in the individual cohorts and potential therapeutic targets for further investigation.

## Methods

Details of the methods of the BALTI-P trial and the associated translational substudy have been published previously.^9^ Patients were randomised to either placebo or inhaled salmeterol preoperatively and postoperatively. At two hospital sites (Queen Elizabeth Hospital Birmingham and Birmingham Heartlands Hospital, UK), patients were recruited to the translational substudy. The VINDALOO trial protocol has been published.^10^ Patients were recruited at Queen Elizabeth Hospital Birmingham and Birmingham Heartlands Hospital, UK, and randomised to either placebo or a single dose of 300 000 IU of vitamin D. In both studies, patients underwent oesophagectomy with care provided as deemed clinically appropriate by the attending surgeons and anaesthetist and followed for their hospital stay.

Databases of the outcomes from the two trials were available for analysis. Smoking status was self-reported in both trials. We collected additional data retrospectively using medical notes, intensive care unit (ICU) charts, electronic patient databases and clinical letters, which provided the preoperative drug history, data for preoperative risk scoring and intraoperative drugs used. The administration of regular medications on the morning of surgery was at the discretion of the attending anaesthetist. In the BALTI-P substudy, patients were excluded if they did not undergo an oesophagectomy with attempted one lung ventilation (OLV). In VINDALOO, only patients who passed the primary endpoint of oesophagectomy with OLV and postoperative PICCO readings were included (consistent with the VINDALOO trial’s analysis).

Differences in the baseline characteristics and perioperative care between trials were assessed. Outcomes for both trials were determined by a clinical endpoints committee. ARDS was defined using the Berlin criteria^11^ for the VINDALOO trial. The BALTI-P trial pre-dates the Berlin criteria, which could not be applied, as applied positive end-expiratory pressure was not recorded. Therefore, we defined ARDS in the BALTI-P trial participants as those with a Pao2:Fio2 (P:F) ratio of 39.9 kPa or below, bilateral chest X-ray infiltrates, attending physician exclusion of cardiogenic dysfunction and requiring invasive ventilation (ventilation with positive end-expiratory pressure of 5 cm H_2_O was standard care in the ICUs involved and non-invasive ventilation was contraindicated in patients following upper gastrointestinal surgery at the time both trials were undertaken).

Continuous variables were subject to normality testing using the Kolmogorov-Smirnov test. For the patients’ baseline data and univariate analysis of perioperative factors, normally distributed continuous variables were analysed with Student’s t-test, non-normally distributed data with the Kruskal-Wallis test and Mann-Whitney U-test and categorical data with the Χ^2^ or Fisher’s exact test as appropriate. Those factors that were significant (P<0.05) were then subject to multivariate analysis. Multivariate analysis of ARDS status was undertaken using forward conditional multivariable binomial logistic regression of the two significant factors in the univariate analysis. Analyses of baseline and univariate data were undertaken using GraphPad Prism V.6.07 for Windows (GraphPad Software, La Jolla, California, USA). Multivariate analyses were performed using SPSS Statistics V.22.0 for Windows.

## Results

Table 1 shows the baseline demographic data from the BALTI-P substudy and VINDALOO groups. Patients in VINDALOO were heavier, received a lower mean tidal volume, received more intravenous fluid, more were on beta-blockers, more received ketamine and dexamethasone and fewer remifentanil and thoracoscopic approach was more common.

**Table 1:** Demographic data from the two trials

	BALTI-P (n=61)	VINDALOO (n=68)	P value
Age (years), median IQR	64 (65–72)	67 (60–72)	0.110
Weight (kg), median IQR	75 (60–84)	77 (68–94)	0.049
Height (cm), median IQR	171 (167–175)	173 (168–177)	0.413
Current smoking	16 (26.7%)	9 (13.4%)	0.075
Histology	Adenocarcinoma 13 (22.8%) Squamous 42 (73.7%) Benign 2 (3.5%)	Adenocarcinoma 58 (85.3%) Squamous 10 (14.7 %) Benign 0 (0.0%)	0.134
Hypertension, n (%)	22 (40.7%)	27 (40.3%)	1.00
Ischaemic heart disease, n (%)	4 (7.40%)	5 (7.46%)	1.00
Diabetes mellitus	5 (9.26%)	8 (11.9%)	0.771
Chronic lung disease	5 (9.26%)	9 (13.4%)	0.574
Venous thromboembolic disease	3 (5.56%)	9 (13.4%)	0.342
Beta blockers, n (%)	4 (7.41%)	16 (23.9%)	0.025
Aspirin, n (%)	9 (16.7%)	11 (16.4%)	1.00
Dihydropyridine	8 (13.1%)	7 (10.3%)	0.784
Statin	11 (20.4%)	22 (32.8%)	0.153
ACE inhibitor or angiotensin II receptor antagonist	11 (20.4%)	13 (19.6%)	1.00
Preoperative haemoglobin (g/dL), mean (SD)	121 (15)	126 (18)	0.080
Mean tidal volume (mL/kg), mean (SD)	6.9 (1.9)	6.1 (1.4)	0.011
Duration of surgery	385 (318–454)	373 (321–419)	0.494
Duration of OLV (min), median (IQR)	155 (130–188)	150 (130–195)	0.794
Fluid administered (mL/kg), median (IQR)	31 (24–46)	41 (30–52)	0.012
Regional anaesthesia used, n (%)	51 (92.7%)	55 (84.6%)	0.254
Remifentanil	13 (24.5%)	5 (8.33%)	0.022
Dexamethasone	8 (15.0%)	20 (66.7%)	0.030
Ketamine	0 (0.0%)	14 (22.2%)	<0.0001
Thoracoscopy	10 (17.9%)	22 (35.5%)	0.039
Laparoscopy	10 (21.7%)	8 (12.7%)	0.455

BALTI-P, Beta Agonist Lung Injury Trial-Prevention; OLV, one lung ventilation; VINDALOO, Vitamin D to Prevent Acute Lung Injury Following Oesophagectomy.

**Figure 1:**
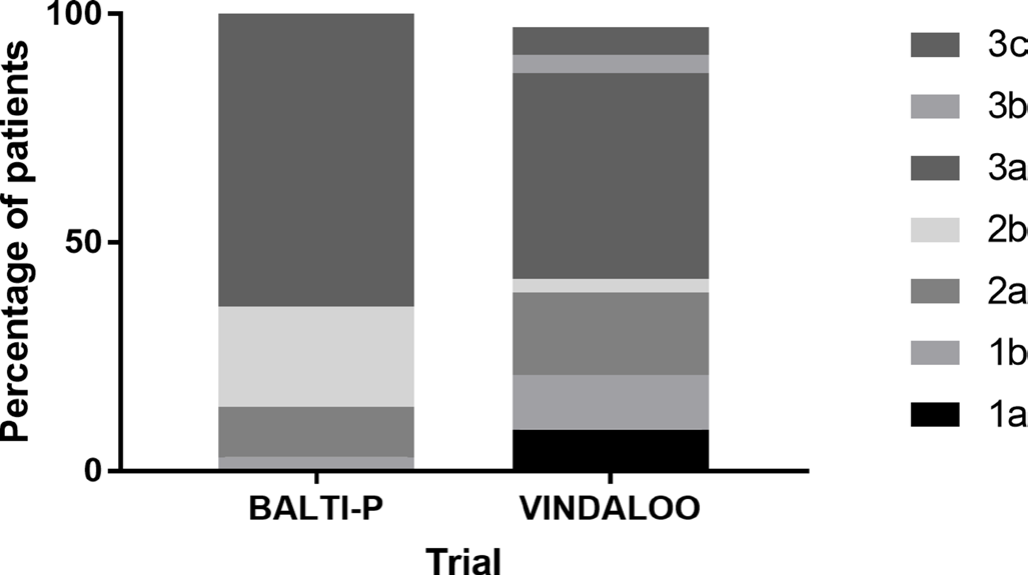
Percentage of patients per stage of oesophageal cancers in the two trials, overall difference P<0.001. BALTI-P Stage 1b n=2, 2a n=6, 2b n=12, 3a n=34, missing/incomplete n=7; VINDALOO 1a n=6, 1b n=8, 2a n=12, 2b n=2, 3a n=29, 3b n=3, 3c n=4, missing/incomplete n=4. BALTI-P, Beta Agonist Lung Injury Trial-Prevention; VINDALOO, Vitamin D to Prevent Acute Lung Injury Following Oesophagectomy.

Staging of malignancy was both more widely distributed and overall higher in the VINDALOO cohort (figure 1). Pre-existing Charlson Index was not different between groups (BALTI-P median 2 (IQR 2–3), VINDALOO 2 (IQR 2–3), P=0.872). Perioperative risk scores were not different between the groups (P-POSSUM Mortality (BALTI-P median 2.4 (IQR 1.9–37) vs VINDALOO 2.4 (IQR 1.5–5.4), P=0.759), P-POSSUM Morbidity (BALTI-P median 8.5 (IQR 4.6–13) vs VINDALOO 8.7 (IQR 6.3–17), P=0.141), O-POSSUM (BALTI-P median 8.5 (IQR 4.6–13) vs VINDALOO 8.7 (IQR 6.3–17), P=0.141)).

To assess risk factors further, the two cohorts were combined and assessed according to ARDS status (table 2). Univariate analysis showed that current smoking and dihydropyridine use were associated with the development of ARDS postoperatively. These variables were then subject to multivariate analysis, which showed that both active smoking (OR 3.91; 95% CI 1.33 to 11.5) and dihydropyridine use (OR 5.34; 95% CI 1.56 to 18.3) remained associated with ARDS risk.

**Table 2:** Comparison of patients with ARDS

Factor	No ARDS (n=108)	ARDS (n=21)	P value
Age, median (IQR)	66 (58–72)	61 (57–70)	0.367
Current smoking, n (%)	17 (16.0%)	8 (38.1%)	0.033
Histology, n (%)			
Adenocarcinoma	85 (80.2%)	15 (78.9%)	0.776
Squamous cell carcinoma	19 (17.9%)	4 (21.1%)	
Benign	2 (1.9%)		
Hypertension, n (%)	40 (38.8%)	9 (50%)	0.439
Ischaemic heart disease, n (%)	9 (8.7%)	0 (0.0%)	0.353
Diabetes mellitus, n (%)	9 (8.7%)	4 (22.2%)	0.103
Lung disease, n (%)	12 (11.7%)	2 (11.1%)	1.00
Venous thromboembolic disease, n (%)	11 (10.7%)	0 (0.0%)	0.367
Weight (kg) median ARDS median (IQR) CDS mean (SD)	75 (65–88)	81 (62–93)	0.485
Height, median (IQR)	173 (167–176)	172 (169–176)	0.915
Haemoglobin, mean (SD)	125 (16)	120 (19)	0.260
Beta-blocker, n (%)	17 (16.7%)	3 (15.8%)	1.00
Dihydropyridine, n (%)	9 (8.3%)	6 (28.6%)	0.0173
Benzothiazepine, n (%)	3 (2.78%)	0 (0.0%)	1.00
Statin, n (%)	28 (27.5%)	5 (26.3%)	1.00
Aspirin, n (%)	16 (15.7%)	4 (21.1%)	0.517
ACE inhibitor or angiotensin II receptor antagonist	20 (19.8%)	4 (21.1%)	1.00
Regional anaesthesia, n (%)	11 (10.9%)	3 (15.8%)	0.464
Remifentanil, n (%)	14 (14.7%)	4 (22.2%)	0.483
Ketamine	12 (12.5%)	2 (11.1%)	1.00
Thoracoscopic approach, n (%)	29 (29.0%)	3 (16.7%)	0.392
Laparoscopic approach, n (%)	84 (83.1%)	17 (94.4%)	0.302

ARDS, acute respiratory distress syndrome.

The effect of these factors on length of stay as a measure of outcome was assessed, as this outcome was collected in both trials. This showed that those patients on dihydropyridines had longer hospital stays (dihydropyridine median 29 days (IQR 17–42), no dihydropyridine 13 days (IQR 10–18), P=0.0007), as did those with diabetes mellitus (diabetes median 25 (IQR 14–39) vs no diabetes 13 (IQR 10–19), P=0.023). There was no difference in length of stay related to smoking (median in never/ex-smokers 13 (IQR 10–23) vs active smokers 15 (IQR 11–20), P=0.73).

## Discussion

Lower tidal volume is now well established in the management of ARDS following the landmark ARDS Clinical Network trial^12^ and there is increasing evidence of its role in intraoperative ventilation.^13 14^ Tidal volumes were lower in the VINDALOO trial, which is likely to represent the increasing adoption of lung protective strategies, including lower tidal volumes, higher positive end-expiratory pressure and permissive hypercarbia.^5^ Whether the reduction of 0.8 mL/kg is clinically significant is not certain, but may be in the context of OLV during oesophagectomy, where less than half the lung volume is subject to intermittent positive pressure ventilation.^15^ This may have played an important role in the change in ARDS incidence. More fluid was administered to the VINDALOO cohort; this might represent a reduction in colloid and increased crystalloid administration and/or more balanced fluid use improving anastomosis perfusion.^6^ Similarly, increasing the use of thoracoscopic techniques and anaesthetic agents with immunomodulatory effects may reduce the inflammatory response to surgery and so the risk of ARDS.^3 5^

This study has indicated that there are two major targets for reduction in postoperative ARDS: cigarette smoking and diyhdropyridines. Smoking has been previously demonstrated to be a risk factor for ARDS,^16 17^ and the fewer current smokers in VINDALOO may have had a marked effect on the ARDS incidence between the two trials. Smoking has been associated with severe perioperative complications in another oesophagectomy cohort.^18^ This work supports the premise of efforts to reduce smoking perioperatively.^19^ Use of nicotine replacement therapy in critical care medicine is controversial, and trials in the perioperative setting are required to ensure safety as well as efficacy.^20^ Evidence of the safety and effectiveness of e-cigarettes and nicotine replacement in the perioperative period also need to be confirmed by randomised trials.^21^

The association between dihydropyridine calcium channel blockers and ARDS was unexpected. ARDS has been reported following dihydropyridine overdose.^22^ Pulmonary oedema following administration of the dihydropyridine nimodipine has been described in the context of subarachnoid haemorrhage.^23^ Potential mechanisms include worsened ventilation-perfusion mismatching due to pulmonary arterial dilatation, reduced cardiac function and pulmonary or inflammatory modulatory effect. Calcium channel blockade has been associated with immunomodulation, although mostly downregulating inflammatory processes.^24–26^ It may be that dihydropyridine use is a marker of worse systemic disease and therefore perioperative risk, although we did not find an association with aspirin, beta-blockers or statins. It would be premature to recommend not using dihydropyridines in the perioperative period, but there is a need for further studies on the effects of concurrent medications on patients undergoing surgery. Such work is underway studying ACE inhibitors (SPACE trial EudraCT 2016-004141-90). Identifying the mechanisms through which dihydropyridines have this effect would also be useful.

A major problem in ARDS prevention trials is identifying a cohort with a high ARDS risk.^8^ Even in the VINDALOO cohort, the ARDS incidence remains higher than that defined by the Lung Injury Prediction Score^27 28^ and the postoperative complication incidence is very high, with the advantages of an initial insult of surgery at a specific time and a defined postoperative care pathway,^3^ which facilitates the conduct of efficacy trials. We believe this work demonstrates that oesophagectomy continues to be a useful model for trialling translational therapeutic and preventative strategies for critical illnesses prior to engaging in larger, more complex and expensive trials.^8^ Examples include the Prevention of Postoperative Pulmonary and Cardiac Complications By Using HMG-CoA Reductase Inhibitor in Patients Undergoing Oesophagectomy (EudraCT Number: 2007-002454-37) and a trial of novel agent GSK2862277 (TFR116341 Trial EudraCT Number: 2014-000643-33).

There are several weaknesses with this investigation. This is a retrospective study and may well be underpowered for some factors, although this work was intended only to be exploratory and hypothesis generating. Much of the data we collected were retrospective and full data were not available for every patient. Additionally, some factors that may be important risk factors for both ARDS and oesophageal cancer, including alcohol consumption,^17^ were not recorded. There were significant differences in potentially important factors in anaesthetic management, discussed above, which potentially complicate comparisons made over time without protocolised surgical or anaesthetic management.

In conclusion, smoking has been associated with higher rates of ARDS following oesophagectomy. The association of dihydropyridines and ARDS requires validation in a larger cohort and mechanistic elucidation. Oesophagectomy continues to have a high risk of ARDS, which continues to offer a useful model for perioperative studies.

## References

[1] Briez N, Piessen G, Torres F, et al Effects of hybrid minimally invasive oesophagectomy on major postoperative pulmonary complications. Br J Surg 2012;99:1547–53. doi:10.1002/bjs.8931 23027071 10.1002/bjs.8931

[2] Tandon S, Batchelor A, Bullock R, et al Peri-operative risk factors for acute lung injury after elective oesophagectomy. Br J Anaesth 2001;86:633–8. doi:10.1093/bja/86.5.633 11575337 10.1093/bja/86.5.633

[3] Howells P, Thickett D, Knox C, et al The impact of the acute respiratory distress syndrome on outcome after oesophagectomy. Br J Anaesth 2016;117:375–81. doi:10.1093/bja/aew178 27440674 10.1093/bja/aew178

[4] Kinugasa S, Tachibana M, Yoshimura H, et al Postoperative pulmonary complications are associated with worse short- and long-term outcomes after extended esophagectomy. J Surg Oncol 2004;88:71–7. doi:10.1002/jso.20137 15499604 10.1002/jso.20137

[5] Carney A, Dickinson M Anesthesia for esophagectomy. Anesthesiol Clin 2015;33:143–63. doi:10.1016/j.anclin.2014.11.009 25701933 10.1016/j.anclin.2014.11.009

[6] Howells P, Bieker M, Yeung J Oesophageal cancer and the anaesthetist. BJA Educ 2017;17:68–73. doi:10.1093/bjaed/mkw037

[7] Sharma S Management of complications of radical esophagectomy. Indian J Surg Oncol 2013;4:105–11. doi:10.1007/s13193-013-0215-1 24426709 10.1007/s13193-013-0215-1 PMC3693150

[8] Proudfoot AG, McAuley DF, Griffiths MJ, et al Human models of acute lung injury. Dis Model Mech 2011;4:145–53. doi:10.1242/dmm.006213 21357760 10.1242/dmm.006213 PMC3046086

[9] Perkins GD, Gates S, Park D, et al The beta agonist lung injury trial prevention. A randomized controlled trial. Am J Respir Crit Care Med 2014;189:674–83. doi:10.1164/rccm.201308-1549OC 24392848 10.1164/rccm.201308-1549OC PMC3983838

[10] Parekh D, Dancer RC, Lax S, et al Vitamin D to prevent acute lung injury following oesophagectomy (VINDALOO): study protocol for a randomised placebo controlled trial. Trials 2013;14:100 doi:10.1186/1745-6215-14-100 23782429 10.1186/1745-6215-14-100 PMC3680967

[11] Ranieri VM, Rubenfeld GD, Thompson BT, Force TADT, et al Acute respiratory distress syndrome: the Berlin definition. JAMA 2012;307:2526–33. doi:10.1001/jama.2012.5669 22797452 10.1001/jama.2012.5669

[12] Brower RG, Matthay MA, Morris A, et al Ventilation with lower tidal volumes as compared with traditional tidal volumes for acute lung injury and the acute respiratory distress syndrome. N Engl J Med 2000;342:1301–8. doi:10.1056/NEJM200005043421801 10793162 10.1056/NEJM200005043421801

[13] Futier E, Constantin JM, Paugam-Burtz C, et al A trial of intraoperative low-tidal-volume ventilation in abdominal surgery. N Engl J Med 2013;369:428–37. doi:10.1056/NEJMoa1301082 23902482 10.1056/NEJMoa1301082

[14] Serpa Neto A, Hemmes SN, Barbas CS, Neto AS, et al Protective versus Conventional Ventilation for Surgery: A Systematic Review and Individual Patient Data Meta-analysis. Anesthesiology 2015;123:66–78. doi:10.1097/ALN.0000000000000706 25978326 10.1097/ALN.0000000000000706

[15] Lohser J, Slinger P Lung injury after one-lung ventilation: A review of the pathophysiologic mechanisms affecting the ventilated and the collapsed lung. Anesth Analg 2015;121:302–18. doi:10.1213/ANE.0000000000000808 26197368 10.1213/ANE.0000000000000808

[16] Iribarren C, Jacobs DR, Sidney S, et al Cigarette smoking, alcohol consumption, and risk of ARDS: a 15-year cohort study in a managed care setting. Chest 2000;117:163–8. 10631215 10.1378/chest.117.1.163

[17] Moazed F, Calfee CS Environmental risk factors for ARDS. Clin Chest Med 2014;35:625–37. 25453414 10.1016/j.ccm.2014.08.003 PMC4255333

[18] Mantziari S, Hübner M, Demartines N, et al Impact of preoperative risk factors on morbidity after esophagectomy: is there room for improvement? World J Surg 2014;38:2882–90. doi:10.1007/s00268-014-2686-9 25002245 10.1007/s00268-014-2686-9

[19] J Wong, F.C Peri-operative cessation of smoking: time for anaesthetists to act. Anaesthesia 2015;70:893–906. 26152251 10.1111/anae.13183

[20] Wilby KJ, Harder CK Nicotine replacement therapy in the intensive care unit: a systematic review. J Intensive Care Med 2014;29:22–30. doi:10.1177/0885066612442053 22513249 10.1177/0885066612442053

[21] Knight-West O, Bullen C E-cigarettes for the management of nicotine addiction. Subst Abuse Rehabil 2016;7:111–8. doi:10.2147/SAR.S94264 27574480 10.2147/SAR.S94264 PMC4993405

[22] Hedaiaty M, Eizadi-Mood N, Sabzghabaee AM Noncardiogenic pulmonary edema after amlodipine overdose without refractory hypotension and bradycardia. Case Rep Emerg Med 2015;2015:1–4. doi:10.1155/2015/546012 10.1155/2015/546012 PMC4436504 26075111

[23] Baker M, Bastin MT, Cook AM, et al Hypoxemia associated with nimodipine in a patient with an aneurysmal subarachnoid hemorrhage. American Journal of Health-System Pharmacy 2015;72:39–43. doi:10.2146/ajhp140196 25511836 10.2146/ajhp140196

[24] Gomes B, Cabral MD, Gallard A, et al Calcium channel blocker prevents T helper type 2 cell-mediated airway inflammation. Am J Respir Crit Care Med 2007;175:1117–24. doi:10.1164/rccm.200607-1026OC 17347497 10.1164/rccm.200607-1026OC

[25] Yasu T, Kobayashi M, Mutoh A, et al Dihydropyridine calcium channel blockers inhibit non-esterified-fatty-acid-induced endothelial and rheological dysfunction. Clin Sci 2013;125:247–55. doi:10.1042/CS20120311 23535137 10.1042/CS20120311

[26] Das R, Burke T, Van Wagoner DR, et al L-type calcium channel blockers exert an antiinflammatory effect by suppressing expression of plasminogen receptors on macrophages. Circ Res 2009;105:167–75. doi:10.1161/CIRCRESAHA.109.200311 19520970 10.1161/CIRCRESAHA.109.200311 PMC2745969

[27] Gajic O, Dabbagh O, Park PK, et al Early identification of patients at risk of acute lung injury: evaluation of lung injury prediction score in a multicenter cohort study. Am J Respir Crit Care Med 2011;183:462–70. doi:10.1164/rccm.201004-0549OC 20802164 10.1164/rccm.201004-0549OC PMC3056224

[28] Trillo-Alvarez C, Cartin-Ceba R, Kor DJ, et al Acute lung injury prediction score: derivation and validation in a population-based sample. Eur Respir J 2011;37:604–9. doi:10.1183/09031936.00036810 20562130 10.1183/09031936.00036810

